# Amplification of the *EGFR* gene can be maintained and modulated by variation of EGF concentrations in *in vitro* models of glioblastoma multiforme

**DOI:** 10.1371/journal.pone.0185208

**Published:** 2017-09-21

**Authors:** Doreen William, Poroshista Mokri, Nora Lamp, Michael Linnebacher, Carl Friedrich Classen, Andreas Erbersdobler, Björn Schneider

**Affiliations:** 1 University Medicine Rostock, Children and Adolescents Hospital, Rostock, Germany; 2 University Medicine Rostock, Institute of Pathology, Rostock, Germany; 3 University Medicine Rostock, Department of General Surgery, Molecular Oncology and Immunotherapy, Rostock, Germany; University of Portsmouth, UNITED KINGDOM

## Abstract

Glioblastoma multiforme (GBM) is the most common and lethal brain tumor in adults. It is known that amplification of the epidermal growth factor receptor gene (*EGFR*) occurs in approximately 40% of GBM, leading to enhanced activation of the EGFR signaling pathway and promoting tumor growth. Although GBM mutations are stably maintained in GBM *in vitro* models, rapid loss of *EGFR* gene amplification is a common observation during cell culture. To maintain *EGFR* amplification *in vitro*, heterotopic GBM xenografts with elevated *EGFR* copy number were cultured under varying serum conditions and EGF concentrations. *EGFR* copy numbers were assessed over several passages by quantitative PCR and chromogenic *in situ* hybridization. As expected, in control assays with 10% FCS, cells lost *EGFR* amplification with increasing passage numbers. However, cells cultured under serum free conditions stably maintained elevated copy numbers. Furthermore, EGFR protein expression positively correlated with genomic amplification levels. Although elevated *EGFR* copy numbers could be maintained over several passages *in vitro*, levels of *EGFR* amplification were variable and dependent on the EGF concentration in the medium. *In vitro* cultures of GBM cells with elevated *EGFR* copy number and corresponding EGFR protein expression should prove valuable preclinical tools to gain a better understanding of *EGFR* driven glioblastoma and assist in the development of new improved therapies.

## Introduction

Glioblastoma multiforme (GBM) is the most common brain tumor in adults with a very dismal prognosis, despite a multimodal, intensive treatment regimen consisting of surgery, radio- and chemotherapy. Therapies are neither particularly effective nor durable with mean survival times of 12–15 months [1–3]. GBMs display a high intratumoral heterogeneity and are characterized by invasive growth into surrounding tissue, making complete resection nearly impossible and favoring development of chemotherapy resistance [3,4].

The EGFR signaling pathway is a prominent regulator of proliferation, growth and survival of mammalian cells [5]. Upon binding of its extracellular ligand EGF or transforming growth factor α (TGFα), EGFR is activated, resulting in enhanced cell proliferation.

Alterations of the *EGFR* gene are considered as frequent driver mutations and are present in approximately 50% of GBM [6,7].

The most common *EGFR* aberration is genomic amplification (40%) [6], often due to extrachromosomal material (double minutes) [8,9], leading to overexpression and enhanced signaling [6]. A multitude of signaling pathway cascades activated by EGFR are associated with GBM progression, e.g. activation of Cyclooxygenase-2 [10,11], K-RAS and AKT signaling [12], and mammalian target of rapamycin (mTOR) together with phosphatidyl-inositol-3-kinase (PI3K) pathways [13–16]. Hence, EGFR or components of its signaling pathway may be promising therapy targets, as shown for tyrosine kinase inhibitors (TKIs) in lung cancer or antibody therapies in colorectal cancer [17,18]. However, substances targeting EGFR or its downstream targets have yet to prove effective in clinical GBM trials [19–21]. Although genomic *EGFR* amplification can be well maintained in *in vivo* models of glioblastoma [22,23], research in a preclinical setting *in vitro* is hampered by the fact that common cell culture models of GBM lack *EGFR* gene amplification [24,25].

Our work shows that this major cell culture disadvantage may be circumvented by culturing the cells under serum free conditions with varying EGF concentrations, leading to the maintenance of *EGFR* gene copy numbers. Although *EGFR* amplification is lost according to EGF concentration, it can subsequently be restored by EGF depletion. This enables further studying of this important signaling pathway in cell lines with the same genetic background and either high or low *EGFR* amplification levels.

## Material & methods

### Cell culture

Cell lines were established from GBM tissue of three different heterotopic patient derived xenograft (PDX) tumors with varying *EGFR* copy numbers [22]. All PDX models were established by implanting GBM tissue received from the operation theater at the department of neurosurgery at the university medicine of Rostock subcutaneously into the flanks of immunodeficient NMRI Foxn^1^ nu mice [22]. Specimen collection was conducted in accordance with the ethics guidelines for the use of human material, approved by the Ethics Committee of the University of Rostock (Reference number: A 2009/34) and with informed written consent from all patients prior to surgery. Prior to cell line establishment, the PDX underwent 2 (xHROG33 and xHROG59) or 5 (xHROG22) serial *in vivo* passages in NMRI Foxn^1^ nu mice. Serial passaging *in vivo* was performed by excision of the subcutaneous xenograft tumors and implanting small pieces of tumor tissue (approximately 3 mm^3^) subcutaneously in the flanks of other NMRI Foxn^1^ nu mice [22]. The obtained GBM PDX tissue was minced using sterile scalpels and passed through a 70 μm cell strainer to obtain single cell suspensions. Cells were divided equally among standard medium control (DMEM/Ham’s F12, 2mM L-Glutamine, 10% FCS) and serum free media cultures (DMEM/Ham’s F12, 2mM L-Glutamine, 1x B-27, 10ng/ml bFGF) with varying amounts of rhEGF (0 ng/ml, 0.5 ng/ml, 1 ng/ml, 1.5 ng/ml, 2 ng/ml, 2.5 ng/ml, 10 ng/ml and 30 ng/ml). Subsequently, all cultures were incubated at 37°C, 5% CO_2_ and 95% relative humidity. Multicellular spheroid cultures were passaged *in vitro* by passing the spheroids through a 70 μM cell strainer. When possible, cultures were sampled at every passage for further analysis.

### Isolation of genomic DNA and PCR analysis

Genomic DNA was isolated using a commercial Kit (Wizard^®^ Genomic DNA Purification Kit, Promega, Mannheim, Germany) following the manufacturer’s instructions. DNA concentration was determined with a spectrophotometer (NanoDrop 1000, Peqlab, Erlangen, Germany). *EGFR* copy numbers were determined by quantitative PCR using 30 ng DNA as template on a StepOne Plus Realtime PCR system (Applied Biosystems, Darmstadt, Germany) with SensiFastSYBR Hi-Rox-Kit (Bioline, Luckenwalde, Germany) (EGFR-for: 5’-TCCCATGATGATCTGTCCCTCACA-3’; EGFR-rev: 5’-CAGGAAAATGCTGGCTGACCTAAG-3’). Commercially available normal Human Genomic DNA (Promega) served as calibrator and the repetitive element LINE1 as endogenous control (LINE1-for: 5’-TGCTTTGAATGCGTCCCAGAG-3’; LINE1-rev: 5’-AAAGCCGCTCAACTACATGG-3’). All reactions were performed in triplicate. The *EGFR* copy number was calculated with the ΔΔCt-algorithm.

Potential cross contamination of the samples with rodent genes was analyzed by PCR (94°C 3min, 35 cycles of 94°C 1min, 65°C 2min, 72°C 1min, final extension at 72°C 4min) using the DFS–Taq polymerase supplied by Bioron (Bioron GmbH, Ludwigshafen, Germany) following manufacturer’s instructions with primers specific for murine *MLH1* (for: 5’-TGTCAATAGGCTGCCCTAGG-3’, rev: 5’-TTTTCAGTGCAGCCTATGCTC-3’) [26]. Human origin of the samples was verified by PCR with primers specific for human cytochrome B (for: 5’- TAGCAATAATCCCCATCCTCCATATTAT-3’, rev: 5’- ACTTGTCCAATGATGGTAAAAGG-3’ [27]) Results were analyzed by gel electrophoresis.

### Paraffin embedding of GBM cells

Cells were harvested and washed with PBS twice. The cells were fixed immediately by resuspending the pellets in 4% buffered formalin (Formafix; Grimm, Torgelow, Germany). Cells were subsequently processed following established standard procedures [28] to form a conglomerate and then embedded in paraffin using the automated Excelsior AS system (Thermo Scientific, Dreieich, Germany) following standard procedures.

### Tissue microarray

Hematoxylin and Eosin (H&E) stained sections of FFPE cell lines were reviewed by an experienced pathologist and regions suitable for analysis were chosen. Tissue microarrays (TMA) were created using a Manual Tissue Arrayer MTA-1 (Beecher Instruments, Sun Prairie, WI, USA) with 1mm diameter punches. From the donor blocks, 3 punches per sample were taken from the selected regions and transferred to an empty acceptor block. The block was heated to 50°C and the correct placement depth of the punches confirmed by microscopic examination. A section from the block was stained with H&E to confirm the presence of a sufficient amount of cells for further analysis.

### Chromogenic *in situ* hybridization

In addition to quantitative PCR analysis, the *EGFR* amplification status was assessed by two-colored chromogenic *in situ* hybridization (2C CISH). 4 μM sections of FFPE tissue samples and TMAs were mounted to coated slides and *EGFR*-specific 2C CISH was performed using the ZytoDot 2C CISH implementation Kit with the ZytoDot 2C SPEC EGFR/CEN 7 Probe (Zytomed Systems, Berlin, Germany) according to manufacturer’s protocols. Processed samples were analyzed by bright field microscopy. Red signals specifically represent the centromere of chromosome 7 for reference and ploidy, whereas green signals are specific for the *EGFR* gene. A cell was considered to carry an *EGFR* amplification if the green / red ratio was >2 or the green signals occurred in clusters.

### Immunohistochemistry

EGFR immunohistochemistry (IHC) was performed using a mouse derived anti-EGFR primary antibody (Zytomed Systems, clone 3G143, dilution 1:200). Slides were processed on an automatic IHC system, AutostainerLink48 (Dako, Hamburg, Germany), according to routine protocols.

### Statistics

Statistical analysis was done using SigmaPlot 10.0 (Systat Software GmbH, Erkrath, Germany). A Kruskal-Wallis one way analysis of variance on ranks in combination with a Tukey test was used for statistical analysis of qPCR data.

## Results

### GBM PDX derived cell lines

**Fig 1:**
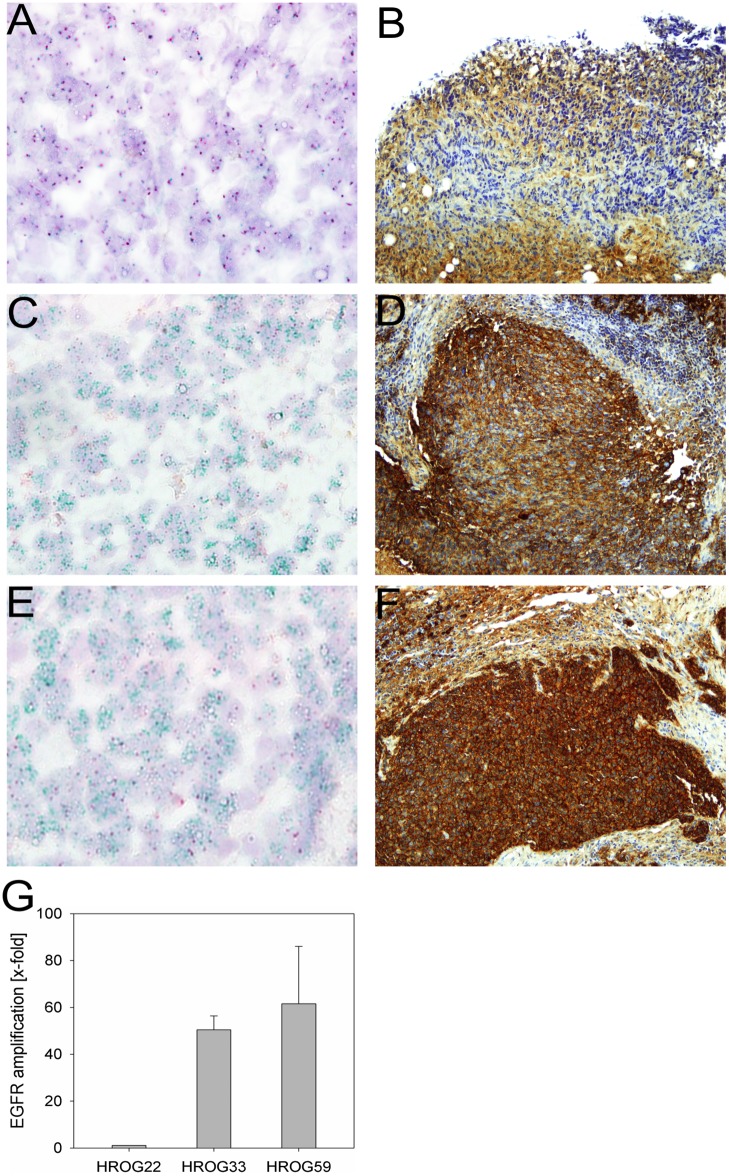
Analysis of *EGFR* amplification by 2C CISH and qPCR and corresponding EGFR protein expression by immunohistochemistry in GBM PDX tissue of HROG22, HROG33 and HROG59. A) 2C CISH of HROG22 (400x magnification), B) EGFR IHC of HROG22 (200x magnification), C) 2C CISH of HROG33 (400x magnification), D) EGFR IHC of HROG33 (200x magnification), E) 2C CISH of HROG59 (400x magnification), F) EGFR IHC of HROG59 (200x magnification); red signals: centromere of chr. 7; green signals: *EGFR* in the 2C CISH analyses; G) qPCR analysis of GBM PDX tissue, error bars represent the standard deviation of triplicate analyses.

In order to identify cell culture conditions, which would allow sustained *EGFR* amplification of GBM cells *in vitro*, cell lines were established from 3 different GBM PDX with varying *EGFR* copy numbers as determined by qPCR and 2C CISH (Fig 1). Prior to cell line establishment, HROG33 and HROG59 PDX underwent 2 serial passages *in vivo*, HROG22 PDX underwent 5 serial passages in NMRI Foxn^1^ nu mice.

HROG33 and HROG59 PDX showed high genomic *EGFR* amplification levels (Fig 1C and 1E), whereas *EGFR* amplification was absent from HROG22 PDX (Fig 1A). The HROG22 PDX also showed a weaker staining in immunohistochemistry analysis of EGFR protein expression, compared to the two *EGFR* amplified PDX, HROG33 and HROG59 (Fig 1B, 1D and 1E). Nevertheless, HROG22 was included in the study to determine if *in vitro* EGF exposure could select for HROG22 cells carrying *EGFR* amplifications. Cell line establishment of xHROG22 and xHROG33 was successful for all conditions tested (serum free with 0, 0.5, 1, 1.5, 2, 2.5, 10, 30 ng/ml EGF and 10% FCS controls). In the case of xHROG59, cell line establishment failed at 2 ng/ml EGF and the 10% FCS control, but was otherwise successful. Contamination of the cell lines with murine DNA was excluded by PCR specific for murine *MLH1* and their human origin was verified by PCR specific for human cytochrome B (S1 Fig, [26,27]). All cell lines established under serum free conditions grew as non-adherent multicellular spheroids regardless of EGF concentration, whereas the 10% FCS controls grew as adherent monolayers. Establishment of spheroid cultures with 10% FCS supplemented medium using anti-adhesive tissue culture dishes failed in all three cases. Although initial spheroid formation was observed, the cells failed to reform spheroids after splitting. Cells from all successful conditions were further analyzed over several passages. In the case of multicellular spheroid cultures, a passage was defined as the time required for single cells growing in suspension to form spheroids. Spheroid formation was considered as complete when they appeared solid in form with no lose single cells attached to the sphere and reached a diameter of approximately 100μM.

### *EGFR* amplification is maintained in serum-free *in vitro* models of GBM and correlates with EGFR protein expression

**Fig 2:**
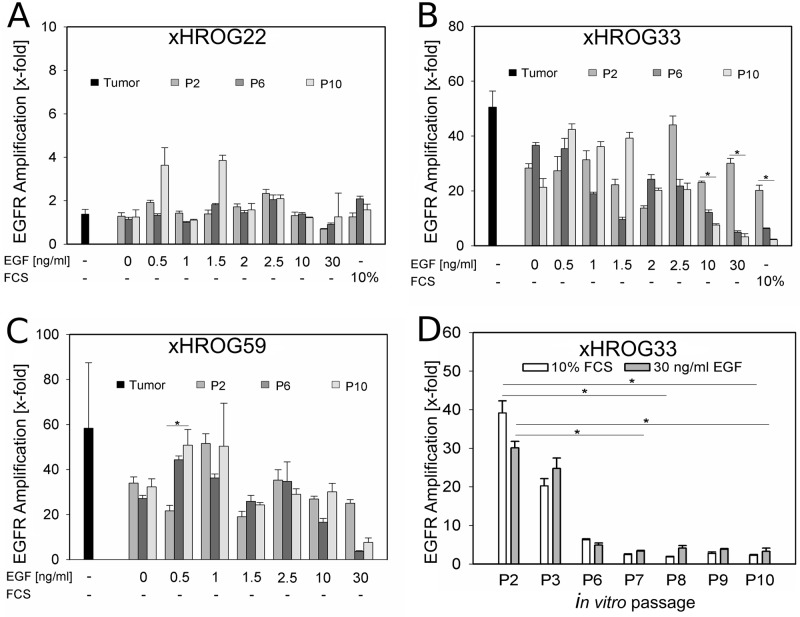
*EGFR* amplification is maintained over several *in vitro* passages, but lost in the 10% FCS control and with high EGF concentrations. qPCR analysis of A) xHROG22, B) xHROG33, C) x HROG59 cell lines grown under all culture conditions at passage 2 (grey bars), passage 6 (dark grey bars) and passage 10 (light grey bars). Black bars represent the respective PDX prior to cell culture establishment. D) qPCR analysis of the 10% FCS control (white bars) and the 30 ng/ml EGF cell line (grey bars) of xHROG33 over several *in vitro* passages. Error bars represent the standard deviation of triplicate analyses. * p<0.05, Tukey test.

Amplification of the *EGFR* gene was analyzed by qPCR over several *in vitro* passages for all culture conditions tested. As expected, *EGFR* amplification was lost in the 10% FCS control of xHROG33 after few passages (Fig 2D, S2 Fig). However, under serum free conditions, *EGFR* amplification was maintained in xHROG33 and xHROG59 over at least 10 *in vitro* passages, although at varying degrees in dependence on the EGF concentration (Fig 2B and 2C, S2 Fig). Of note, GBM cells cultured with high amounts of EGF (30 ng/ml) showed the same rapid loss of *EGFR* amplification as the 10% FCS control (Fig 2D). In case of xHROG22, no marked differences in *EGFR* amplification were observed under all conditions applied with copy numbers below four (Fig 2A, S2 Fig).

**Fig 3:**
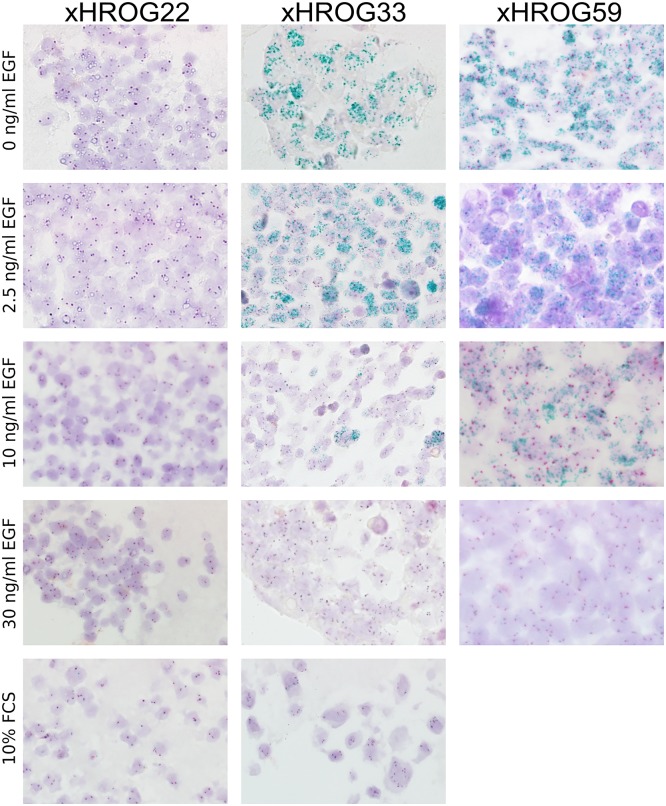
2C CISH analysis confirms qPCR data of *EGFR* amplification. 2C CISH analyses of xHROG22, xHROG33 and xHROG59 cell lines established under different conditions as indicated at passage 10 of cell culture; 400x magnification.

To further confirm the qPCR data, cells at passage 10 were fixed in formalin and embedded in paraffin for *EGFR* specific CISH analyses (Fig 3). The 2C CISH analyses showed a high number of *EGFR* gene copies in xHROG33 cell lines cultured serum free with low amounts of EGF, whereas culture at 10 ng/ml EGF was accompanied by lowered amplification levels. Parity of chromosome 7 specific and *EGFR* specific signals indicating the loss of *EGFR* amplification was observed in xHROG33 cells cultured with 30 ng/ml EGF in the medium and the 10% FCS control (Fig 3). Similar results were obtained in xHROG59 (Fig 3). As expected, there was no increase of *EGFR* signal in xHROG22 (Fig 3). Thus, the 2C CISH results are well in line with the results obtained by qPCR analyses of *EGFR* amplification.

**Fig 4:**
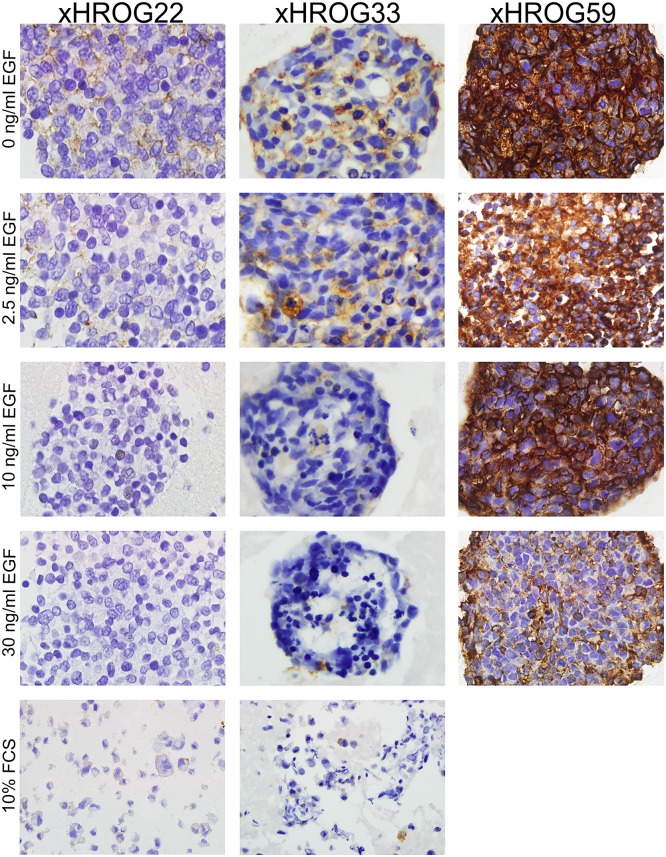
EGFR protein expression is concordant with genomic *EGFR* amplification. EGFR immunohistochemistry staining of paraffin embedded xHROG22, xHROG33 and xHROG59 cells grown under different conditions as indicated at passage 10 of cell culture; 200x magnification.

In order to determine if *EGFR* amplification levels also result in increased EGFR protein expression, TMA sections of embedded GBM cells were subjected to EGFR IHC staining (Fig 4). In all cases, the obtained results accorded with the 2C CISH and qPCR data (Figs 2 and 3).

### Loss of *EGFR* amplification can be restored by EGF withdrawal

**Fig 5:**
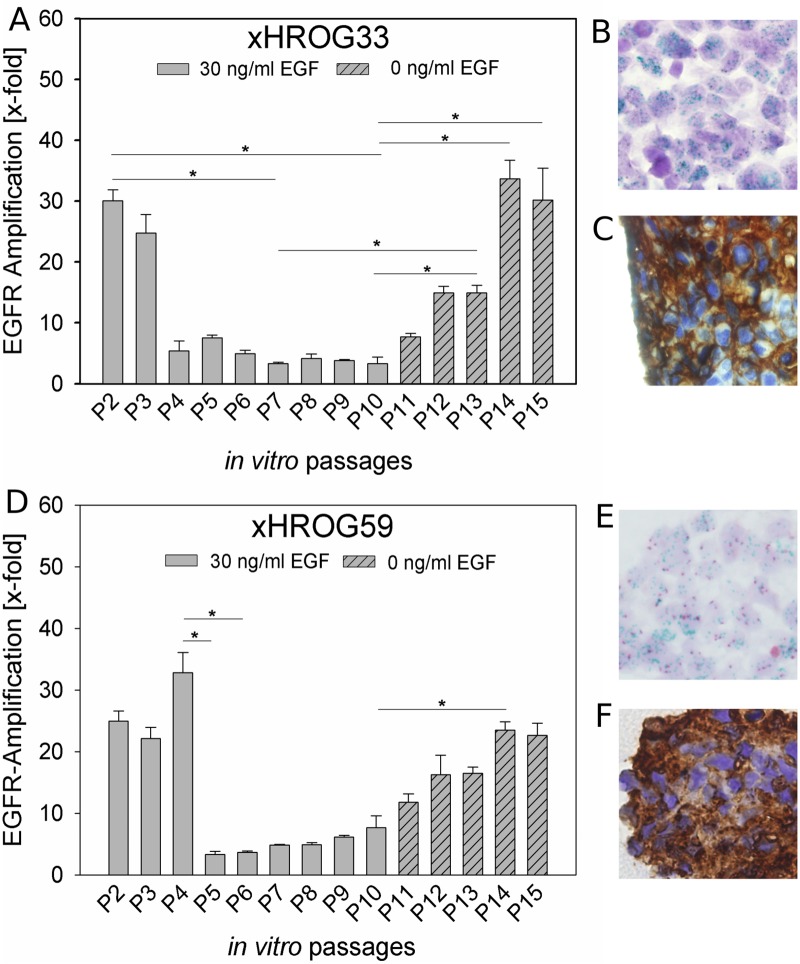
EGF withdrawal restores *EGFR* amplification. A and D) qPCR analysis of xHROG33 (A) and xHROG59 (D) cultured with 30 ng/ml EGF until passage 10 (grey bars) and without EGF for 5 additional passages (dashed bars), respectively. Error bars represent the standard deviation of triplicate analyses; B and E) 2C CISH analysis of paraffin embedded samples of xHROG33 (B) and xHROG59 (E) after 5 passages post EGF withdrawal (P15), C and F) EGFR immunohistochemistry staining of paraffin embedded samples of xHROG33 (C) and xHROG59 (F) after 5 passages post EGF withdrawal (P15). 400x magnification. * p<0.05, Tukey test.

Genomic amplification of *EGFR* appears to be dependent on the EGF concentration present in the medium (Figs 2 and 3, S2 Fig). We observed a decrease in *EGFR* amplification with 10 ng/ml EGF in xHROG33 and xHROG59 and, additionally, for xHROG33 an almost complete loss of *EGFR* amplification with 30 ng/ml EGF approaching the level of the 10% FCS control. In both cases, after culture with 30 ng/ml EGF for 10 passages, EGF was withdrawn and *EGFR* amplification was analyzed by qPCR, 2C CISH and EGFR IHC staining after 5 *in vitro* passages post EGF depletion. *EGFR* amplification and protein expression were gradually restored after EGF withdrawal (Fig 5). In the case of the 10% FCS control of xHROG33, the cells failed to adapt to the change from 10% FCS to serum free without EGF followed by cell death.

## Discussion

Glioblastoma multiforme remains a lethal brain tumor despite intensified treatment strategies, highlighting the need for more effective therapies [1,2]. Nearly half of GBM cases display a genomic amplification of *EGFR* and subsequent hyperactivation of the PI3K/AKT/mTOR signaling pathways [5,13,14]. However, preclinical *in vitro* studies are complicated by the rapid loss of genomic *EGFR* amplification under standard cell culture conditions [24,25]. GBM *in vitro* models with stable *EGFR* amplification would represent an experimental system more faithfully representing the original tumor. The *in vitro* models in this study were established from GBM PDX instead of directly patient derived GBM tissue. We confirmed the human origin of all PDX models as well as their resemblance to the primary GBM in a previous study [22]. All PDX models were histologically analyzed by a neuropathologist and found to fulfill requirements for a GBM diagnosis [22]. Additionally, the human origin of the established cell lines was verified (S1 Fig), suggesting that the findings of this study would likely be translatable to directly patient derived *in vitro* models.

Although the exact doubling times of the established cell lines were not determined, we did not observe distinct differences in cell proliferation between cell lines cultivated with low EGF and high EGF concentrations. However, other studies observed higher proliferation rates with primary GBM cell cultures established under high EGF concentrations compared to cell lines of the same origin cultured without EGF supplementation [29].

We observed spontaneous spheroid formation in all cell lines under all applied serum free conditions independent on the EGF concentration in the media. Since the culture method applied in this study (serum free media supplemented with B27 and FGF) is often used to enrich cancer stem-like cells (CSCs) of GBM we hypothesized that the spontaneous spheroid formation we observed under those conditions may be attributable to CSCs [30,31].

In the case of the *EGFR* unamplified xHROG22, obtaining *EGFR* amplified cells under the applied *in vitro* conditions proved unsuccessful. It is likely that an initial event to establish extrachromosomal *EGFR* amplification, like unscheduled DNA synthesis and replication or inverted duplications, occurs during tumorigenesis [32]. In case of HROG22 such an event apparently did not occur, suggesting that this tumor is not dependent on *EGFR* amplification but rather on other mechanisms and pathways. It was not possible to induce *EGFR* amplification *in vitro* under the conditions applied in this study, suggesting that the xHROG22 cell line is similarly not dependent on *EGFR* amplification.

Two cell lines analyzed in this study (xHROG33 and xHROG59) stably maintained *EGFR* amplification under low EGF conditions ranging from 0 to 2.5 ng/ml over at least 10 *in vitro* passages in long-term cell culture (12 to 15 months). *EGFR* amplification levels decreased rapidly with higher EGF concentrations in both cases.

These results are well in line with previous studies, demonstrating maintained *EGFR* amplification under serum free conditions without EGF supplementation as well as decreased *EGFR* amplification with high EGF concentrations in primary GBM cell lines [23,29,33]. Hence, *EGFR* amplification levels appear to be directly influenced by the EGF concentration *in vitro*.

However, Schulte *et al*. reported an almost complete loss of *EGFR* amplification in primary GBM cell lines cultivated with 10 ng/ml EGF [29]. In the case of xHROG33 a decrease in *EGFR* amplification was observed in qPCR results, but 2C CISH analysis confirmed the presence of *EGFR* amplified cells. However, in the case of xHROG59 cells established under serum free conditions with 10 ng/ml EGF, we did not observe a major decrease of *EGFR* amplification in qPCR, 2C CISH or EGFR IHC analysis.

In case of xHROG33 we observed a rapid decrease of *EGFR* amplification in the 10% FCS control similar to the serum free culture with 30 ng/ml EGF. Although the morphology of the cells is different–spheroids in the serum free culture and adherent monolayer with 10% FCS–we deemed it unlikely that the morphology plays a significant role for decreased *EGFR* amplification of the 10% FCS cell line of xHROG33. Would the loss of *EGFR* amplification be attributable to cell attachment, we would have expected no similarly rapid decrease of *EGFR* amplification in the spheroid culture of xHROG33. Although not further investigated, we hypothesize that the variety of growth factors present in FCS has a similar effect on the *EGFR* amplification of the cells as the supplementation of high EGF amounts.

Our qPCR and 2C CISH data are concordant with EGFR protein expression as determined by IHC staining of EGFR, indicating that the genomic amplification is functional.

Furthermore, restoration of genomic *EGFR* amplification after high *in vitro* EGF-induced loss was possible by complete EGF withdrawal in both cases analyzed (xHROG33 and xHROG59). Previous studies demonstrated restoration of *EGFR* amplification in primary GBM cell lines by reducing EGF concentrations from 20 ng/ml to 5 ng/ml EGF [33]. However, other studies failed to restore *EGFR* amplification after withdrawal of EGF in primary GBM cell lines with high EGF-induced *EGFR* amplification loss [29,31]. Interestingly, similar restoration effects have been described in a model of EGFR inhibitor resistance [34]. Nathanson *et al* found extrachromosomal *EGFR* present in untreated GBM cells, which was lost under erlotinib treatment, but restored subsequently to drug removal [34]. Although in our study the withdrawal of EGF and not discontinued treatment with an EGFR inhibitor lead to restoration of *EGFR* amplification *in vitro*, the underlying mechanism might be similar. A marker chromosome including *EGFR* positive homogeneous staining regions (HSR) might serve as *EGFR* reservoir, enabling tumor cells to regain extrachromosomal *EGFR* amplification in response to microenvironmental stimuli like discontinued EGFR inhibitor exposure or, possibly, EGF withdrawal [34–36].

However, this was not further investigated in our study and thus remains speculative.

In sum, the GBM *in vitro* models described here stably maintain *EGFR* amplification under low EGF conditions. Furthermore exposure of cell lines that were initially derived from the same PDX tissue showed decreased *EGFR* amplification when high amounts of EGF were supplemented. Thus, these *in vitro* models are a useful tool for in depth analysis of EGFR expression level dependent effects in an isogenic background.

## Supporting information

S1 Fig PCR analysis of murine *MLH1* and human specific *cytochrome B*. A) Analysis of gDNA pools of xHROG22 (xH.22), xHROG33 (xH.33) and xHROG59 (xH.59) at passage 2 (P2) and passage 10 (P10). Ctr M: DNA derived from mouse tail tissue as positive control for murine *MLH1* (~350bp), Ctr H: DNA derived from a GBM cell line established from patient derived GBM tissue B) Verification of human origin of the cell lines by human specific *cytochrome B* PCR (~130bp product).
(TIF)

S2 Fig qPCR analysis of *EGFR* amplification of all successfully established cell lines. Tukey test, * p<0.05.
(TIF)

## References

[1] Dolecek TA, Propp JM, Stroup NE, Kruchko C. CBTRUS Statistical Report: Primary Brain and Central Nervous System Tumors Diagnosed in the United States in 2005–2009. Neuro-Oncology 2012;14: 1–49. 10.1093/neuonc/nos218 PMC3480240 23095881

[2] Pollo B. Neuropathological diagnosis of brain tumours. Neurological Sciences 2011;32: 209–11. 10.1007/s10072-011-0802-2 22009244

[3] Stupp R, Hegi ME, Mason WP, van den Bent MJ, Taphoorn MJ, Janzer RC et al Effects of radiotherapy with concomitant and adjuvant temozolomide versus radiotherapy alone on survival in glioblastoma in a randomised phase III study: 5-year analysis of the EORTC-NCIC trial. The Lancet Oncology 2009;10: 459–66. doi: 10.1016/S1470-2045(09)70025-7 19269895 10.1016/S1470-2045(09)70025-7

[4] Lima FR, Kahn SA, Soletti RC, Biasoli D, Alves T, da Fonseca AC et al Glioblastoma: Therapeutic challenges, what lies ahead. Biochimica et Biophysica Acta (BBA)—Reviews on Cancer 2012;1826: 338–49. 22677165 10.1016/j.bbcan.2012.05.004

[5] Thorne AH, Zanca C, Furnari F. Epidermal growth factor receptor targeting and challenges in glioblastoma. Neuro Oncol. 2016;18(7): 914–8. doi: 10.1093/neuonc/nov319 26755074 10.1093/neuonc/nov319 PMC4896544

[6] Furnari FB, Fenton T, Bachoo RM, Mukasa A, Stommel JM, Stegh A, et al Malignant astrocytic glioma: genetics, biology, and paths to treatment. Genes Dev. 2007;21(21): 2683–2710. doi: 10.1101/gad.1596707 17974913 10.1101/gad.1596707

[7] Shinojima N, Tada K, Shiraishi S, Kamiryo T, Kochi M, Nakamura H, et al Prognostic value of epidermal growth factor receptor in patients with glioblastoma multiforme. Cancer Res. 2003 10 15;63(20): 6962–70. 14583498

[8] Muleris M, Almeida A, Dutrillaux AM, Pruchon E, Vega F, Delattre JY et al Oncogene amplification in human gliomas: a molecular cytogenetic analysis. Oncogene 1994;9(9): 2717–22. 8058336

[9] Nikolaev S, Santoni F, Garieri M, Makrythanasis P, Falconnet E, Guipponi M, et al Extrachromosomal driver mutations in glioblastoma and low-grade glioma. Nat Commun. 2014;5: 5690 doi: 10.1038/ncomms6690 25471132 10.1038/ncomms6690 PMC4338529

[10] Lo HW, Cao X, Zhu H, Ali-Osman F. Cyclooxygenase-2 is a novel transcriptional target of the nuclear EGFR-STAT3 and EGFRvIII-STAT3 signaling axes. Mol Cancer Res. 2010;8(2): 232–245. doi: 10.1158/1541-7786.MCR-09-0391 20145033 10.1158/1541-7786.MCR-09-0391 PMC2824777

[11] Shono T, Tofilon PJ, Bruner JM, Owolabi O, Lang FF. Cyclooxygenase-2 expression in human gliomas: prognostic significance and molecular correlations. Cancer Res. 2001;61(11): 4375–4381. 11389063

[12] Lyustikman Y, Momota H, Pao W, Holland EC. Constitutive activation of Raf-1 induces glioma formation in mice. Neoplasia 2008;10(5): 501–10. 18472967 10.1593/neo.08206 PMC2373912

[13] Parsons DW, Jones S, Zhang X, Lin JC, Leary RJ, Angenendt P, et al An integrated genomic analysis of human glioblastoma multiforme. Science 2008;321(5897): 1807–12. doi: 10.1126/science.1164382 18772396 10.1126/science.1164382 PMC2820389

[14] Cancer Genome Atlas Research Network. Comprehensive genomic characterization defines human glioblastoma genes and core pathways. Nature 2008;455(7216): 1061–8. doi: 10.1038/nature07385 18772890 10.1038/nature07385 PMC2671642

[15] Akhavan D, Cloughesy TF, Mischel PS. mTOR signaling in glioblastoma: lessons learned from bench to bedside. Neuro Oncol. 2010;12(8): 882–889. doi: 10.1093/neuonc/noq052 20472883 10.1093/neuonc/noq052 PMC2940679

[16] Nicholas MK, Lukas RV, Jafri NF, Faoro L, Salgia R. Epidermal growth factor receptor—mediated signal transduction in the development and therapy of gliomas. Clin Cancer Res. 2006;12(24): 7261–70. doi: 10.1158/1078-0432.CCR-06-0874 17189397 10.1158/1078-0432.CCR-06-0874

[17] McCoach CE, Jimeno A. Osimertinib, a third-generation tyrosine kinase inhibitor targeting non-small cell lung cancer with EGFR T790M mutations. Drugs Today (Barc). 2016;52(10): 561–568. 27910964 10.1358/dot.2016.52.10.2541343

[18] Kircher SM, Nimeiri HS, Benson AB 3rd. Targeting Angiogenesis in Colorectal Cancer: Tyrosine Kinase Inhibitors. Cancer J. 2016;22(3): 182–9. doi: 10.1097/PPO.0000000000000192 27341596 10.1097/PPO.0000000000000192

[19] Lee EQ, Kaley TJ, Duda DG, Schiff D, Lassman AB, Wong ET, et al A Multicenter, Phase II, Randomized, Noncomparative Clinical Trial of Radiation and Temozolomide with or without Vandetanib in Newly Diagnosed Glioblastoma Patients. Clin Cancer Res. 2015;21(16): 3610–8. doi: 10.1158/1078-0432.CCR-14-3220 25910950 10.1158/1078-0432.CCR-14-3220 PMC4790106

[20] Chen C, Ravelo A, Yu E, Dhanda R, Schnadig I. Clinical outcomes with bevacizumab-containing and non-bevacizumab-containing regimens in patients with recurrent glioblastoma from US community practices. J Neurooncol. 2015;122(3): 595–605. doi: 10.1007/s11060-015-1752-y 25773061 10.1007/s11060-015-1752-y PMC4436682

[21] Westphal M, Heese O, Steinbach JP, Schnell O, Schackert G, Mehdorn M, et al A randomised, open label phase III trial with nimotuzumab, an anti-epidermal growth factor receptor monoclonal antibody in the treatment of newly diagnosed adult glioblastoma. Eur J Cancer 2015;51(4): 522–32. doi: 10.1016/j.ejca.2014.12.019 25616647 10.1016/j.ejca.2014.12.019

[22] William D, Mullins CS, Schneider B, Orthmann A, Lamp N, Krohn M, et al Optimized creation of glioblastoma patient derived xenografts for use in preclinical studies. J Transl Med. 2017;15(1): 27 doi: 10.1186/s12967-017-1128-5 28183348 10.1186/s12967-017-1128-5 PMC5301415

[23] Stockhausen MT, Broholm H, Villingshøj M, Kirchhoff M, Gerdes T, Kristoffersen K, et al „Maintenance of EGFR and EGFRvIII expressions in an in vivo and in vitro model of human glioblastoma multiforme. Exp Cell Res. 2011;317(11): 1513–26. doi: 10.1016/j.yexcr.2011.04.001 21514294 10.1016/j.yexcr.2011.04.001

[24] Humphrey PA, Wong AJ, Vogelstein B, Friedman HS, Werner MH, Bigner DD, et al Amplification and expression of the epidermal growth factor receptor gene in human glioma xenografts. Cancer Res 1988;48: 2231–8. 3258189

[25] Pandita A, Aldape KD, Zadeh G, Guha A, James CD. Contrasting in vivo and in vitro fates of glioblastoma cell subpopulations with amplified EGFR. Genes Chromosomes Cancer 2004;39: 29–36. doi: 10.1002/gcc.10300 14603439 10.1002/gcc.10300

[26] Maletzki C, Beyrich F, Hühns M, Klar E, Linnebacher M. The mutational profile and infiltration pattern of murine MLH1-/- tumors: concurrences, disparities and cell line establishment for functional analysis. Oncotarget 2016;7(33): 53583–53598. doi: 10.18632/oncotarget.10677 27447752 10.18632/oncotarget.10677 PMC5288207

[27] Matsuda H, Seo Y, Kakizaki E, Kozawa S, Muraoka E, Yukawa N. Identification of DNA of human origin based on amplification of human-specific mitochondrial cytochrome b region. Forensic Sci Int. 2005;152(2–3): 109–14. doi: 10.1016/j.forsciint.2004.07.019 15978336 10.1016/j.forsciint.2004.07.019

[28] Dijkstra JR, Opdam FJ, Boonyaratanakornkit J, Schönbrunner ER, Shahbazian M, Edsjö A, et al Implementation of formalin-fixed, paraffin-embedded cell line pellets as high-quality process controls in quality assessment programs for KRAS mutation analysis. J Mol Diagn. 2012;14(3): 187–91. doi: 10.1016/j.jmoldx.2012.01.002 22414609 10.1016/j.jmoldx.2012.01.002

[29] Schulte A, Gunther HS, Martens T, Zapf S, Riethdorf S, Wulfing C et al Glioblastoma stem-like cell lines with either maintenance or loss of high-level EGFR amplification, generated via modulation of ligand concentration. Clin Cancer Res 2012; 18(7): 1901–13. doi: 10.1158/1078-0432.CCR-11-3084 22316604 10.1158/1078-0432.CCR-11-3084

[30] Ernst A, Hofmann S, Ahmadi R, Becker N, Korshunov A, Engel F, et al Genomic and expression profiling of glioblastoma stem cell-like spheroid cultures identifies novel tumor-relevant genes associated with survival. Clin Cancer Res. 2009;15(21): 6541–50. doi: 10.1158/1078-0432.CCR-09-0695 19861460 10.1158/1078-0432.CCR-09-0695

[31] Soeda A, Inagaki A, Oka N, Ikegame Y, Aoki H, Yoshimura S-I et al Epidermal growth factor plays a crucial role in mitogenic regulation of human brain tumor stem cells. J Biol Chem 2008;283(16): 10958–66. doi: 10.1074/jbc.M704205200 18292095 10.1074/jbc.M704205200

[32] Stark G. R., Debatisse M., Giulotto E. & Wahl G. M. Recent progress in understanding mechanisms of mammalian DNA amplification. Cell 1989;57: 901–908 2661014 10.1016/0092-8674(89)90328-0

[33] Mazzoleni S, Politi LS, Pala M, Cominelli M, Franzin A, Sergi Sergi L et al Epidermal growth factor receptor expression identifies functionally and molecularly distinct tumor-initiating cells in human glioblastoma multiforme and is required for gliomagenesis. Cancer Res 2010;70(19): 7500–13. doi: 10.1158/0008-5472.CAN-10-2353 20858720 10.1158/0008-5472.CAN-10-2353

[34] Nathanson DA, Gini B, Mottahedeh J, Visnyei K, Koga T, Gomez G, et al Targeted therapy resistance mediated by dynamic regulation of extrachromosomal mutant EGFR DNA. Science. 2014;343(6166): 72–6. doi: 10.1126/science.1241328 24310612 10.1126/science.1241328 PMC4049335

[35] Turner KM, Deshpande V, Beyter D, Koga T, Rusert J, Lee C, et al Extrachromosomal oncogene amplification drives tumour evolution and genetic heterogeneity. Nature 2017;543(7643): 122–125. doi: 10.1038/nature21356 28178237 10.1038/nature21356 PMC5334176

[36] Windle B., Draper B. W., Yin Y. X., O’Gorman S. & Wahl G. M. A central role for chromosome breakage in gene amplification, deletion formation, and amplicon integration. Genes Dev. 1991;5: 160–174. 1995414 10.1101/gad.5.2.160

