# Supplementary figures and images for: Amplification of the EGFR gene can be maintained and modulated by variation of EGF concentrations in in vitro models of glioblastoma multiforme
Source: PLoS One. 2017 Sep 21;12(9):e0185208. doi: 10.1371/journal.pone.0185208 (PMC5608330; doi:10.1371/journal.pone.0185208)

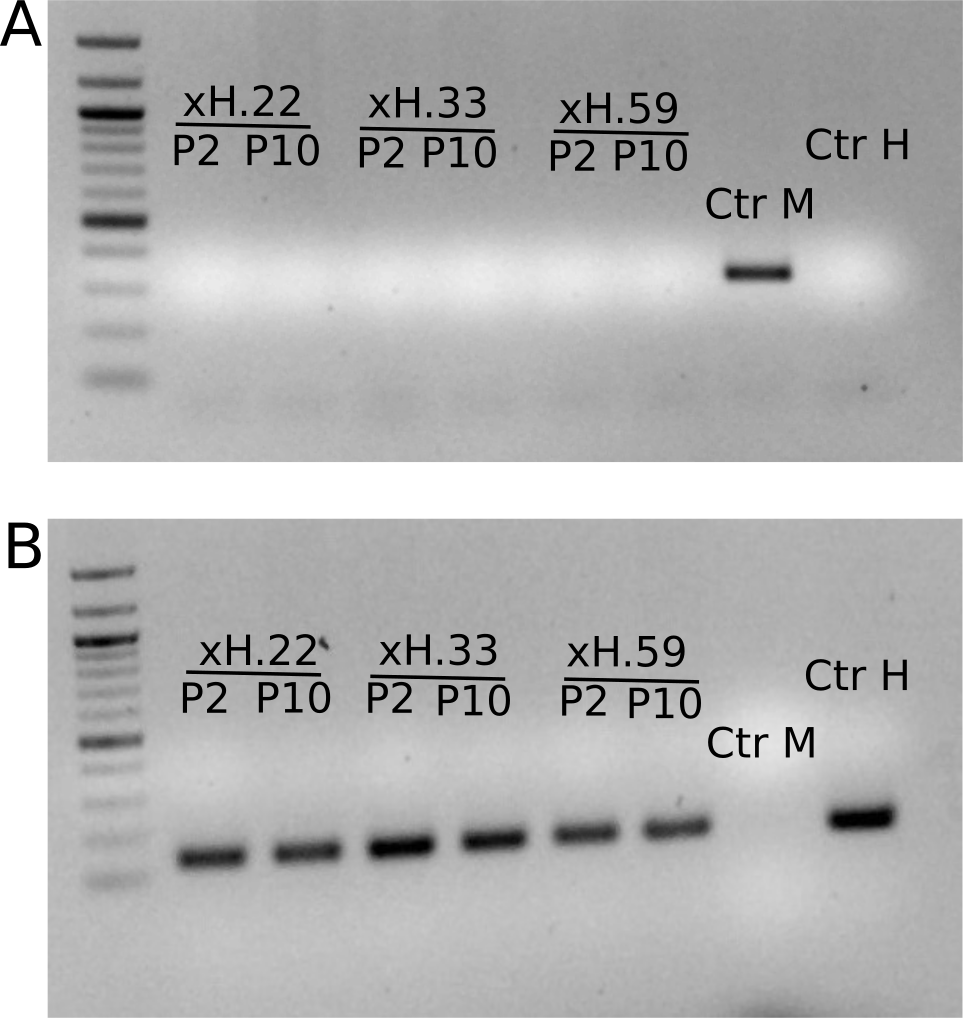

Supplement: S1 Fig — A) Analysis of gDNA pools of xHROG22 (xH.22), xHROG33 (xH.33) and xHROG59 (xH.59) at passage 2 (P2) and passage 10 (P10). Ctr M: DNA derived from mouse tail tissue as positive control for murine MLH1 (~350bp), Ctr H: DNA derived from a GBM cell line established from patient derived GBM tissue B) Verification of human origin of the cell lines by human specific cytochrome B PCR (~130bp product). (TIF) [file pone.0185208.s001.tif]

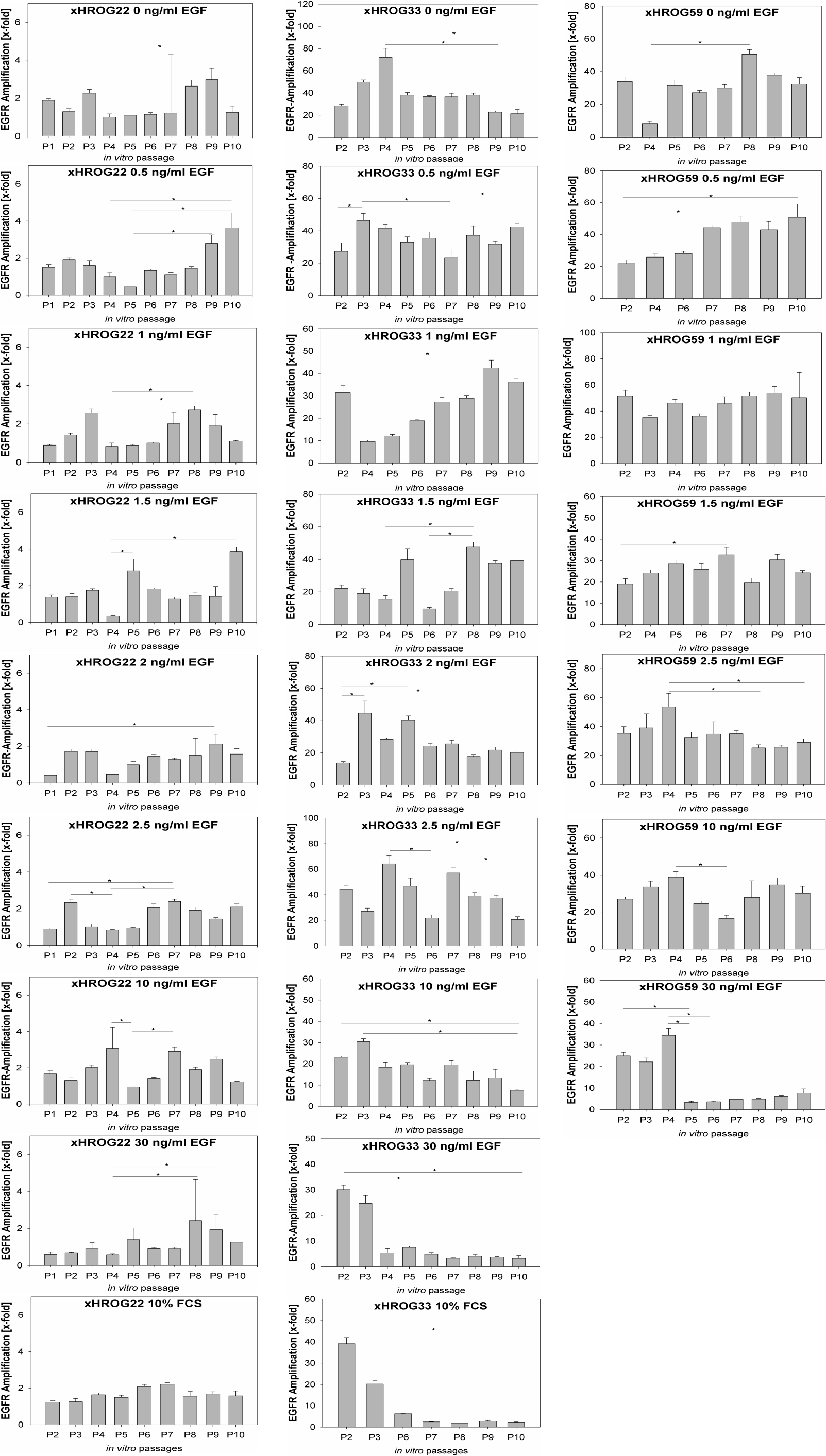

Supplement: S2 Fig — Tukey test, * p<0.05. (TIF) [file pone.0185208.s002.tif]
